# A deep learning approach for automatic 3D segmentation of hip cartilage and labrum from direct hip MR arthrography

**DOI:** 10.1038/s41598-025-86727-z

**Published:** 2025-02-07

**Authors:** Malin Kristin Meier, Ramon Andreas Helfenstein, Adam Boschung, Andreas Nanavati, Adrian Ruckli, Till D. Lerch, Nicolas Gerber, Bernd Jung, Onur Afacan, Moritz Tannast, Klaus A. Siebenrock, Simon D Steppacher, Florian Schmaranzer

**Affiliations:** 1 Department of Orthopedic Surgery, University Hospital, Inselspital Bern, University of Bern, Freiburgstrasse, Bern, 3010 Switzerland; 2 Department of Orthopaedic Surgery and Traumatology, Fribourg Cantonal Hospital, University of Fribourg, Chemin des Pensionnats, Villars-sur-Glâne, Fribourg, 1752 Switzerland; 3 Department of Diagnostic, Interventional and Pediatric Radiology, Inselspital, Bern University Hospital, University of Bern, Freiburgstrasse, Bern, 3010 Switzerland; 4 Department of Radiology, Boston Children’s Hospital, 300 Longwood Ave, Boston, MA 02215 USA; 5 Faculty of Medicine, Department of Radiology, Balgrist University Hospital, University of Zürich, Forchstrasse 340, Zürich, 8008 Switzerland

**Keywords:** Translational research, Cartilage

## Abstract

The objective was to use convolutional neural networks (CNNs) for automatic segmentation of hip cartilage and labrum based on 3D MRI. In this retrospective single-center study, CNNs with a U-Net architecture were used to develop a fully automated segmentation model for hip cartilage and labrum from MRI. Direct hip MR arthrographies (01/2020-10/2021) were selected from 100 symptomatic patients. Institutional routine protocol included a 3D T1 mapping sequence, which was used for manual segmentation of hip cartilage and labrum. 80 hips were used for training and the remaining 20 for testing. Model performance was assessed with six evaluation metrics including Dice similarity coefficient (DSC). In addition, model performance was tested on an external dataset (40 patients) with a 3D T2-weighted sequence from a different institution. Inter-rater agreement of manual segmentation served as benchmark for automatic segmentation performance. 100 patients were included (mean age 30 ± 10 years, 64% female patients). Mean DSC for cartilage was 0.92 ± 0.02 (95% confidence interval [CI] 0.92–0.93) and 0.83 ± 0.04 (0.81–0.85) for labrum and comparable (*p* = 0.232 and 0.297, respectively) to inter-rater agreement of manual segmentation: DSC cartilage 0.93 ± 0.04 (0.92–0.95); DSC labrum 0.82 ± 0.05 (0.80–0.85). When tested on the external dataset, the DSC was 0.89 ± 0.02 (0.88–0.90) and 0.71 ± 0.04 (0.69–0.73) for cartilage and labrum, respectively.The presented deep learning approach accurately segments hip cartilage and labrum from 3D MRI sequences and can potentially be used in clinical practice to provide rapid and accurate 3D MRI models.

## Introduction

Evaluation of hip cartilage and labrum is one of the main tasks of hip MRI in patients with hip deformities such as femoroacetabular impingement or hip dysplasia. These deformities result in altered hip biomechanics and increased stress to cartilage and labrum^1^. Standard evaluation of hip cartilage and labrum is based on morphologic 2D MRI and focusses on assessment of morphologic damage as well as gross anatomy. This is only an approximation to the actual 3D anatomy. Reliable information about 3D anatomy of hip cartilage and labrum has great potential to aid surgeons in choosing the appropriate surgical procedure^2^. In addition, standard 2D MRI is limited to detection of advanced cartilage damage while biochemically sensitive MRI such as delayed gadolinium enhanced MRI of cartilage (dGEMRIC) can be used as a more sensitive and quantitative cartilage biomarker, thereby guiding the surgeon in the question of whether hip joint preserving or replacement surgery should be performed^3,4^.

In daily clinical practice, a detailed morphologic 3D analysis of hip cartilage and labrum as well as the assessment of dGEMRIC indices is not routinely feasible due to the laborious task of manual segmentation. Manual segmentation of combined hip labrum and cartilage based on MRI is challenging, requires > 3 h for one case and has to be performed by expert readers, i.e. orthopedic surgeons or musculoskeletal radiologist with several years of experience in hip MRI. Several attempts have been made to perform this task for the hip cartilage automatically, including 3D deformable models^5^ and multitemplate based label fusion techniques^6^. However, these attempts yielded only moderate results. In comparison to the knee, automatic segmentation of the hip cartilage seems to be a more challenging task, due to its naturally thinner structure. More recently, deep learning-based techniques have shown potential to increase segmentation performance of hip cartilage^7,8^. However, these studies are based on a small sample size (< 25 patients) and they do not include the labrum. Since the cartilage and labrum act as one biomechanic entity, combined segmentation of hip labrum and cartilage it the logical conclusion. To the best of our knowledge, fully automated cartilage and labrum segmentation to create 3D models from MRI using deep learning has not yet been explored. Automatically segmented 3D MRI models of the hip labrum and cartilage enable a level of detailed 3D anatomical description previously unattainable. Few studies performed on selected 2D images have indicated that the labrum size and morphology reflect the underlying pathomechanism in the hip joint, e.g. labrum hypertrophy in hip instability^9,10^. 2D analyses only represent an approximation of the complex 3D anatomy of the hip joint. Automatic segmentation of hip cartilage and labrum paves way for a deeper understanding of the 3D anatomical differences between normal and pathological hip morphologies. In addition, it might facilitate identification of the underlying hip pathology, thereby potentially improving surgical decision making in young patients at risk for premature osteoarthritis of the hip. Most importantly, it opens the doors for patient-specific 3D anatomical analysis in the future. The aim of this study was to develop and validate a deep learning approach for fully automatic MRI based 3D models of hip labrum and cartilage based on direct MR arthrography of the hip.

## Methods

### Study design

The study protocol was approved by the local ethics committee (Kantonale Ethikkomission Bern, Switzerland, KEK 2022 − 00618) with a waiver for informed consent and performed in accordance with appropriate guidelines. We performed a retrospective feasibility study to develop and validate a deep learning approach for automated 3D segmentation of hip cartilage and labrum based on a direct hip MR arthrography using a recently introduced 3D T1 cartilage mapping sequence (MP2RAGE = Magnetization-prepared 2 Rapid Gradient-Echo) and a high-resolution balanced steady state free precession sequence (TrueFISP = true fast imaging with steady state precession). This study was performed in accordance with all relvant guidelines and regulations including the declaration of Helsinki.

### Data

**Fig. 1:**
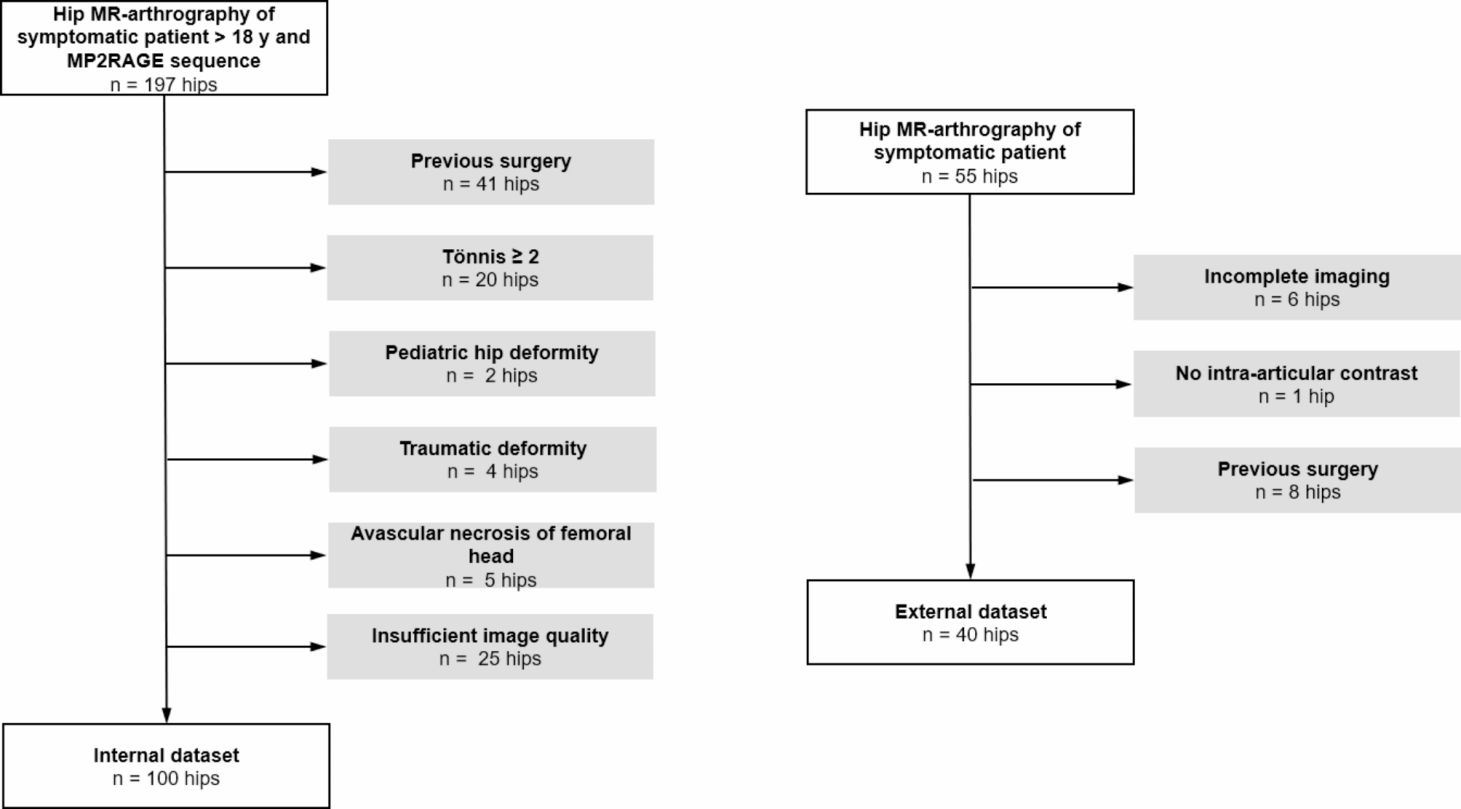
Patient inclusion and exclusion. Flowchart of patient inclusion and exclusion.

The internal dataset was developed by querying the picture archiving and communication system of the radiology department of the Bern University hospital for direct hip MR-arthrograms between January 2020 and October 2021. Inclusion criteria were a symptomatic hip deformity, patient aged > 18 years and a complete MRI scan according to the institutional routine protocol including a 3D T1 MP2RAGE sequence (Table 1). This resulted in a consecutive series of 197 patients. Exclusion criteria were posttraumatic deformity, previous hip surgery, pediatric hip deformities and insufficient image quality such as motion artefacts or extra-articular contrast agent. This resulted in a total of 100 patients in the internal dataset (Fig. 1).

**Table 1:** Sequence protocol.

Sequence	Repetition Time (ms)	Echo Time (ms)	Inversion times (ms)	Acquisitionmatrix	Reconstruction matrix	FOV (mm)	Flip angle	Slice Thickness (mm)	Bandwidth (Hz/Px)	Image orientation
3D T1 MP2RAGE	5000	3.4	400/ 2500	192 × 192	384 × 384	173	4, 5	1	240	axial-oblique
3D T2-w TrueFISP	10.95	4.73	n.a.	384 × 384	384 × 384	170	28	1	63.67	axial-oblique

*MP2RAGE* magnetization prepared two rapid gradient echo^15^;* TrueFISP *  true fast imaging with steady-state progression.

The external dataset was developed by querying the picture archiving and communication system of the radiology department of the Bern University hospital for direct hip MR-arthrograms between December 2021 and September 2022. Inclusion criteria were symptomatic hip deformity, patient aged > 18 years and a complete MRI scan according to the institutional routine protocol including a T2-weighted 3D TrueFISP sequence (Table 1). This resulted in a consecutive series of 55 patients. The same exclusion criteria as for the internal data set were applied. This resulted in a total of 40 patients in the external dataset (Fig. 1).

### MR image acquisition

For both internal and external data sets, direct hip MR arthrography was performed under fluoroscopic guidance with injection of 1—2 ml of iodinated contrast agent (Iopamidol 200 mg/ml; Iopamiro 200; Bracco), 2-5 ml of local anaesthetic (ropivacaine hydrochloride; 2 mg/ml; Ropinaest; GebroPharma), and 15-20 ml of diluted MR contrast agent (gadopentetate dimeglumine, 2mmol/l, Magnevist; Bayer Healthcare). Multiplanar proton density (PD) weighted turbo spin echo (TSE) images of the hip were performed in coronal-, sagittal- and radial image orientation. The internal data set was acquired at a 3T unit (Magnetom Skyra, Siemens Healthineers) and included an axial-oblique 3D T1 MP2RAGE sequence which was used for manual and automatic segmentation as well as for postcontrast T1 mapping of the hip joint (dGEMRIC). The external dataset was acquired at a 1.5T unit (Magnetom Aera, Siemens Healthineers) and included an axial-oblique 3D T2-w TrueFISP sequence which was used for manual and automatic segmentation.

### Ground truth segmentation

**Fig. 2:**
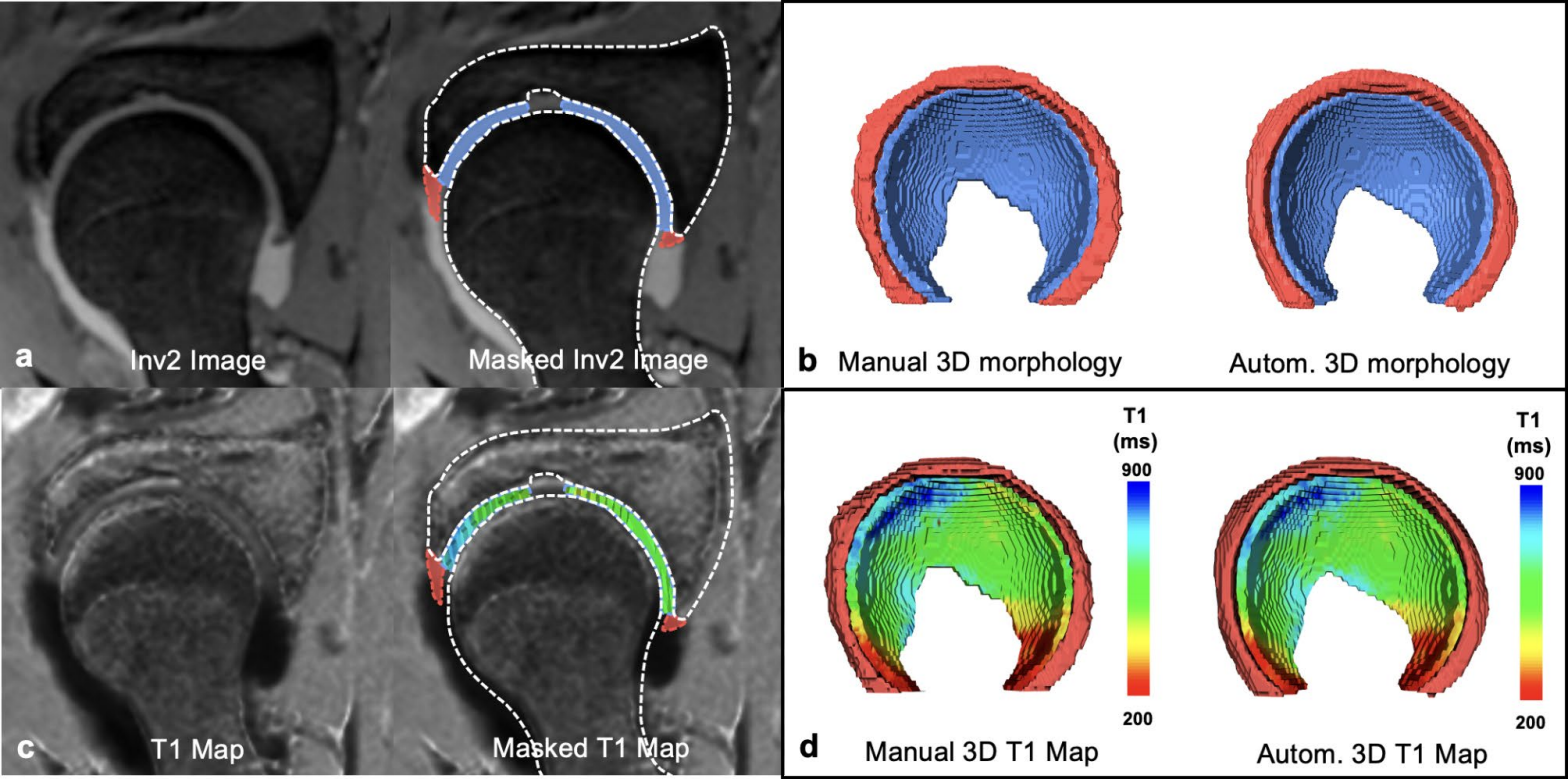
Manual segmentation workflow of internal dataset. Manual segmentation workflow for hip cartilage and labrum on the internal data set. (**a**) Raw image (Inv2) of the 3D T1 magnetization prepared 2 rapid acquisition gradient echoes (MP2RAGE) sequence were used for manual segmentation of hip cartilage (blue) and labrum (red). (**b**) Corresponding manual and automatic 3D morphologic models. (**c**) Masks of cartilage and labrum were then applied to the co-registered T1 map for (**d**) 3D visualization of post-contrast T1 relaxation time (dGEMRIC) using a voxel wise color-graded scale. Dice Similarity Coefficient between manual and automatic segmentation of this particular example was 0.95 for cartilage and 0.87 for labrum. 3D models were visualized using a custom-made software application for automatic segmentation and visualization^8^.

**Fig. 3:**
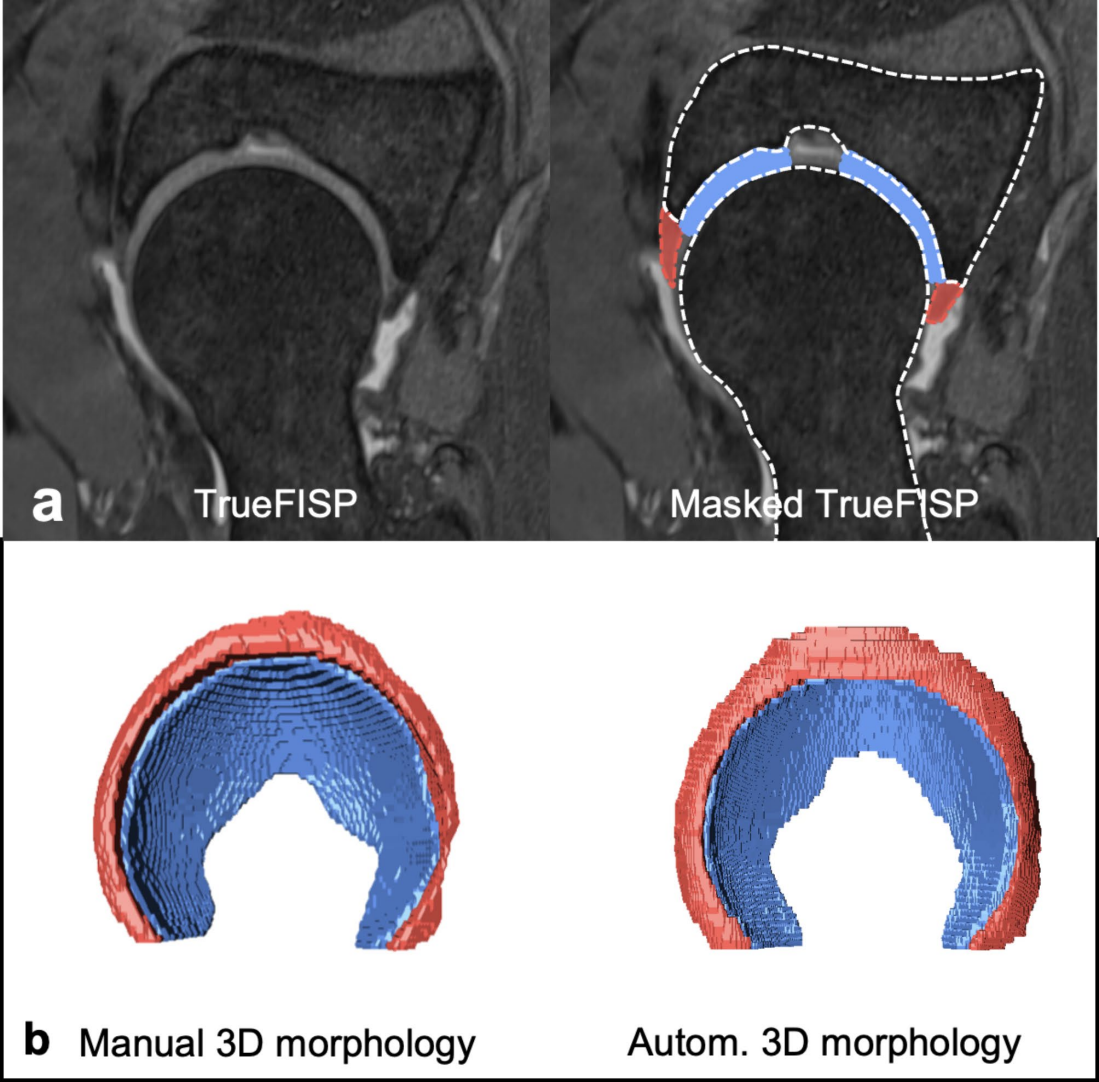
Manual segmentation workflow of external dataset. Workflow of manual segmentation of hip cartilage and labrum for the external data set. (**a**) The T2 weighted true fast imaging with steady state precession (TrueFISP) sequence was used for manual segmentation of hip cartilage (blue) and labrum (red). (**b**) Corresponding 3D visualization of cartilage (blue) and labrum (red) morphology. Dice Similarity Coefficient of this particular example was 0.92 for cartilage and 0.78 for labrum. 3D models were visualized using a custom-made software application for automatic segmentation and visualization^8^.

Manual segmentations were performed in a standardized multistep-approach with a commercially available software (Amira 6.1, FEI, Hilsboro, Oregon, USA) using the 3D T1 MP2RAGE (internal data set) and 3D T2-w TrueFISP (external data set) in the axial-oblique plane, without any preprocessing steps. Since the T1 maps do not provide adequate image contrast for segmentation the raw data acquired with the second inversion pulse was used (Inv2 images). These images yield improved image contrast of the chondro-labral structures and bone. Manual segmentations were performed by two residents (MKM, AB), each with 3 years of experience in hip imaging, and checked by a radiologist (FS) with 8 years of experience in hip imaging, i.e. each slice was checked and corrected if needed. Using a standardized approach for segmentation (Figs. 2 and 3), the osseous acetabulum and proximal femur were first labelled using a threshold-assisted method. In a second step, the femoroacetabular cartilage layers were labelled extending from the medial (acetabular fossa) to the lateral border using the acetabular rim as reference. Finally, based on the created models of hip cartilage and the acetabular rim, the labrum was labelled from its attachment at the acetabular rim and cartilage to its apex (Figs. 2 and 3). To assess inter- and intra-rater variability, a subset of randomly chosen 20 patients of the internal data set was additionally segmented by a second reader (AB) and repeated 6 weeks later by one of the readers (AB) to serve as a benchmark for automatic segmentation accuracy.

### Data partitions

The overall sample size of this study included 140 patients, 100 patients in the internal and 40 patients in the external data set (Fig. 1). Of the internal data set, the first 80 patients were consecutively selected for model training (internal training set). The remaining 20 patients were excluded from training and used as unseen data for model testing (internal testing set).

For the external validation, the 40 patients were split in two external datasets. The first external testing set included 20 unseen cases (external set 1 test). To investigate if model performance can be improved with additional model training, we used the external test set 1 for additional training and the “external set 2 retraining” for testing.

### Model

The pipeline contained initial cropping, segmentation and metric calculation^8^. As preprocessing step, the image volume was automatically cropped to 80 × 200 × 200 voxels around the femoral head center to increase efficiency and to reduce background complexity for the convolutional neural network. The automatic cropping was performed using a landmark detection algorithm, based on a U-Net architecture with heatmap regression and a receptive field width of 140 pixels^8,11^. No image reformation was performed. The external sets were resampled to the same voxel spacing and cropped to the same size. For segmentation of three-dimensional structure of hip cartilage and labrum, the 3D U-net architecture^12^ was used. For generation of the 3D U-net, the training, evaluation, and prediction, the self configurating nnU-net framework from Isensee et al.^13^ was applied.

The nnU-Net prevents overfitting through data augmentation, adaptive patch sampling, and an ensemble of five networks trained during cross-validation. This ensemble improves generalization, achieving similar or better results on the test set than the validation set. Early stopping also helps by halting training when validation performance stabilizes, preventing excessive fitting.

**Fig. 4:**
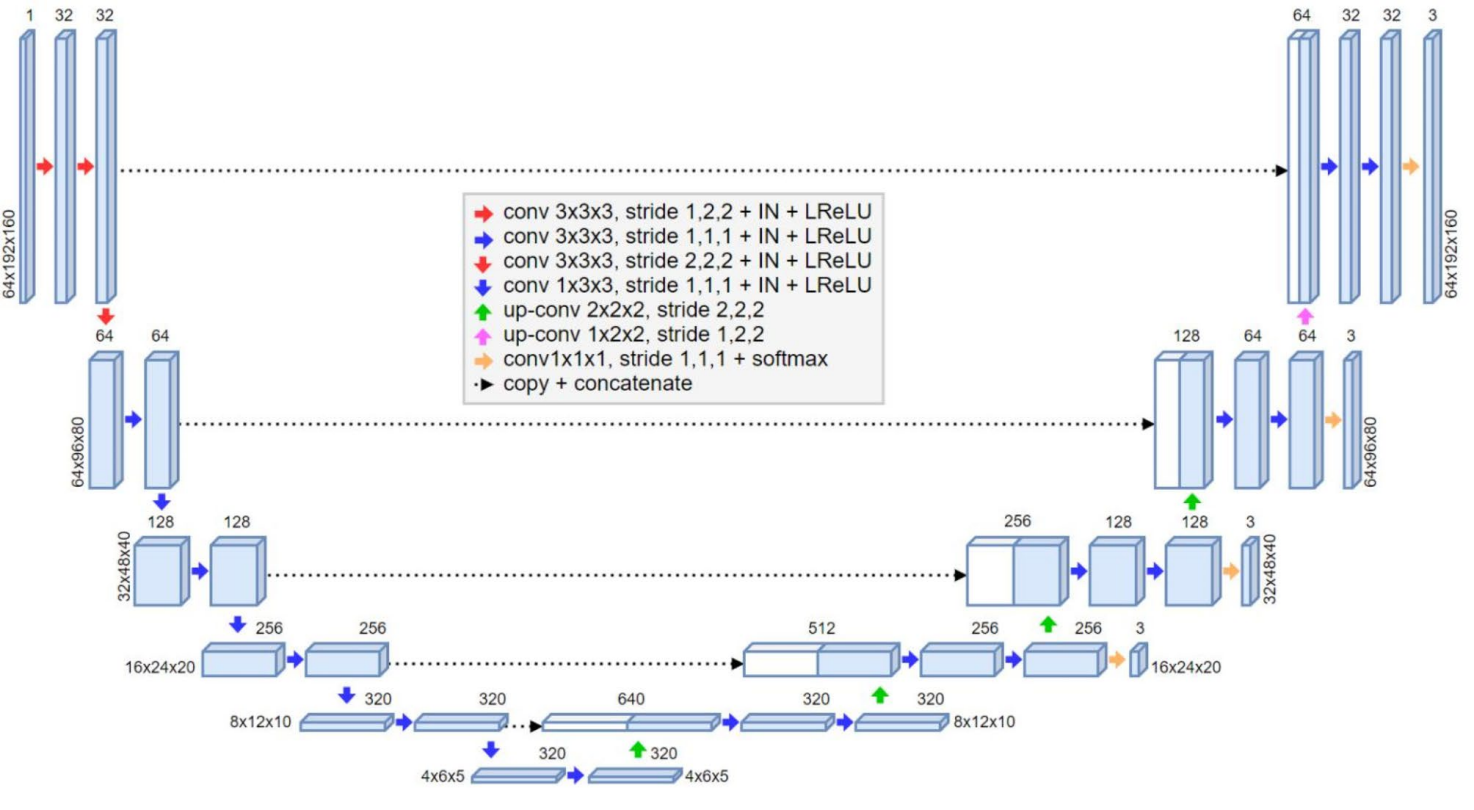
3D U-net architecture. 3D U-net architecture with a single channel input patch of 64 × 192 × 160 voxels. Each blue box corresponds to a multi-channel feature map. The number of channels is denoted on top of the box. The size of the volumetric patch is constant per layer and indicated on the left and the right. The white boxes represent copied feature maps. The arrows denote the different operations. The first layer is based on a pseudo 2D configuration. The deeper layers are based on a 3D configuration. The output segmentation feature maps, including the maps for the deep supervision, consists of 3 channels, each related to the background, cartilage, or labrum label.

The deep learning network architecture included encoder and decoder paths, which were connected via skip connections, as well as a bottle neck at the lowest layer (Fig. 4). The base building block was a convolution followed by an instance normalization (IN) layer and a leaky rectified linear Unit (LReLU) (negative slope, 0.01) as activation function. Two consecutive building blocks were used for each resolution step. The first layer in the encoder and decoder paths operated only on the axial planes in a pseudo 2D configuration to create or extract from isotropic image features. Downsampling was implemented as strided convolution. Upsampling was implemented as transposed convolution. The initial feature map consisted of 32 channels and doubled with each downsampling operation, limited to a maximum of 320 channels. The patch size was 64 × 192 × 160 voxels. Segmentation masks were generated with a 1 × 1 × 1 convolution and a SoftMax layer followed by an ArgMax operation for background, cartilage or labrum tissue. The network was trained with deep supervision; additional auxiliary losses were added in the decoder to all but the two lowest resolutions. This allowed the gradients to be injected deeper into the network, facilitating the training of all layers in the network^13^.

### Training

The network was trained on the internal data set (*n* = 80) for 60 epochs from scratch with random weights. One epoch was defined as iteration over 250 mini batches. A mini batch consisted of two patches due to limited GPU memory. Stochastic gradient descent with Nesterov momentum (*µ* = 0.99) and an initial learning rate of 0.01 was used for learning network weights. The learning rate was decayed throughout the training, (1 − epoch*/*epoch_max_)^0.9^. The loss function was the sum of cross-entropy and Dice loss.

The framework was trained 5-fold for a cross-fold validation to evaluate the performance on the validation sets and to define the post processing steps. nnU-Net empirically opted for ‘non-largest component suppression’ as a post-processing step if performance gains were measured^13^. The final network was an ensemble to increase the generalizability, where the final SoftMax layer was a result of the averaged 5 subnetworks.

### Evaluation

The model performance was evaluated by the following metrics: Dice similarity coefficient (DSC), average symmetric surface distance (ASSD), precision, recall, absolute and relative differences in volume and dGEMRIC indices.

### Code availability statement

The code for preprocessing, training and evaluation is publicly available on GitHub. https://doi.org/10.5281/zenodo.14316889

### Statistical analysis

Data was tested for normal distribution using Kolmogorov Smirnov test. For comparison between data sets Kruskall Wallis Test with Dunn’s correction for multiple comparisons was used for continuous parameters and Chi-Square Test for binary data. Difference in dGEMRIC indices of manual and automatic cartilage segmentation was assessed with Wilcoxon Rank Test for paired data. All statistical tests were conducted at the two-sided 5% significance level using GraphPad Prism (Version 9.5, GraphPad Software)

## Results

### Data

Demographic characteristics are presented in Table 2. The study cohort included multiple femoral and acetabular deformities (Table 2). Internal training and test data set did not differ regarding morphology (Table 2).

**Table 2:** Patient characteristics and radiographic parameters of the data sets.

	Internal training set	Internal testing set	External set 1 test	External set 2 retraining	*P* Value Overall	*P* Value Internal training set vs. internal testing set	*P* Value Internal test set vs. external set 1 test	*P* Value External set 1√ vs. external set 2 retraining
Patient characteristics								
Number of patients / hips ()	80	20	20	20	-	-	-	-
Female (% of hips)	64	65	50	55	0.600			
Mean age (years)	31 ± 10; 29–33	28 ± 10; 23–31	33 ± 12; 27–39	38 ± 10; 33–43	0.017	0.817	0.827	0.601
Radiographic parameters								
–Joint degeneration								
Tönnis grade 1 (% of hips)	39	60	55	65	0.086	-	-	-
Acetabular morphology								
Lateral center edge (LCE) angle (°)	30 ± 9; 28–32	29 ± 10; 25–34	32 ± 5; 30–34	29 ± 5; 27–31	0.518	-	-	-
Acetabular index (°)	3 ± 6; 2–4	3 ± 7; -1–6	1 ± 5; -1–3	4 ± 4; 1–6	0.299	-	-	-
Retroversion index (%)	16 ± 19; 12–20	13 ± 18; 5–22	19 ± 15; 13–26	14 ± 13; 9–20	0.631	-	-	-
Femoral morphology								
Alpha angle (°)	64 ± 6; 63–66	60 ± 10; 55–65	62 ± 6; 59–65	58 ± 7; 55–61	0.001	0.067	> 0.999	0.346
Femoral torsion* (°)	30 ± 12; 27–32	27 ± 7; 24–31	26 ± 13; 20–33	30 ± 7; 26–33	0.487	-	-	-

Continuous parameters are presented as mean ± standard deviation and 95% confidence; * measured according to Murphy et al.^20^.

### Model performance

The non-largest component suppression as post processing step, had an influence of 1 part per million on the DSC in all data sets and was negligible for both cartilage and labrum.

### Internal test set

Mean DSC for cartilage was 0.92 ± 0.02 (95% confidence interval [CI] 0.92–0.93) and 0.83 ± 0.04 (0.81–0.85) for labrum (Tables 3 and 4). Mean volume of manually/automatically segmented cartilage was 7195 ± 1587 (6452–7938) / 7176 ± 1600 (6427–7925)mm^3^, resulting in a mean relative difference in cartilage volume of − 4.1 ± 3.5 (2.5–5.7)%. Mean volume of the manually/automatically segmented labrum was 2720 ± 642 (2420–3020) / 2477 ± 702 (2149–2806)mm^3^, resulting in a mean relative difference in volume of 13.4 ± 12.7 (7.4–19.3)%.

**Table 3:** Evaluation metrics of model segmentation performance of hip cartilage with comparison between internal and external data sets.

	Internal test set	External set 1 test	External set 2 retraining	*P* Value Internal test set vs. external test set 1	*P* Value External set 1 test vs. external set 2 retraining	*P* Value Internal test set vs. external set 2 set	Overall *P* Value
DSC ()	0.92 ± 0.02; 0.92–0.93	0.89 ± 0.02; 0.88–0.90	0.92 ± 0.02; 0.91–0.93	< 0.001	< 0.001	> 0.999	< 0.001
ASSD (mm)	0.22 ± 0.05; 0.20–0.24	0.37 ± 0.10; 0.32–0.42	0.26 ± 0.10; 0.22–0.30	< 0.001	0.001	0.665	< 0.001
Precision ()	0.92 ± 0.03; 0.91–0.94	0.94 ± 0.03; 0.92–0.95	0.91 ± 0.04; 0.90–0.93	-	-	-	0.076
Recall ()	0.93 ± 0.03; 0.91–0.94	0.85 ± 0.05; 0.83–0.87	0.93 ± 0.03; 0.91–0.94	< 0.001	< 0.001	> 0.999	< 0.001
Absolute difference in volume (mm^3^)	282 ± 231; 174–390	901 ± 477; 678–1124	402 ± 288; 268–537	< 0.001	0.001	0.868	< 0.001
Relative difference in volume (%)	4.1 ± 3.5; 2.5–5.7	10.6 ± 5.2; 8.1–13.0	4.9 ± 3.7; 3.2–6.7	< 0.001	0.003	> 0.999	< 0.001
Absolute difference in dGEMRIC index (ms)	5.4 ± 4.6; 3.3–7.5	-	-	-	-	-	-
Relative difference in dGEMRIC index (%)	1.0 ± 0.9; 0.6–1.5	-	-	-	-	-	-

Continuous parameters are presented as mean ± standard deviation with 95% confidence interval; DSC = dice similarity coefficient; ASSD = average symmetric surface distance, dGEMRIC = delayed gadolinium enhanced MRI of cartilage.

**Table 4:** Evaluation metrics of model segmentation performance of hip labrum with comparison between internal and external data sets.

	Internal test set		External set 1 test		External set 2 retraining		*P* Value Internal test set vs. external test set 1		*P* Value External set 1 test vs. external set 2 retraining		*P* Value Internal test set vs. external set 2 set		Overall *P* Value
DSC ()	0.83 ± 0.04; 0.81–0.85		0.71 ± 0.04; 0.69–0.73		0.77 ± 0.05; 0.74–0.79		< 0.001		0.020		0.009		< 0.001
ASSD (mm)	0.35 ± 0.10; 0.30–0.39		0.64 ± 0.20; 0.55–0.74		0.48 ± 0.16; 0.41–0.56		< 0.001		0.016		0.036		< 0.001
Precision ()	0.79 ± 0.08; 0.75–0.82		0.72 ± 0.06; 0.69–0.74		0.79 ± 0.09; 0.75–0.83		< 0.001		0.053		0.005		< 0.001
Recall ()	0.78 ± 0.03; 0.85–0.88		0.71 ± 0.07; 0.67–0.74		0.76 ± 0.10; 0.71–0.81		0.008		0.082		> 0.999		0.008
Absolute difference in volume (mm^3^)	296 ± 280; 166–427		212 ± 240; 100–324		409 ± 410; 217–601		-		-		-		0.130
Relative difference in volume (%)	13.4 ± 12.7; 7.4–19.3		9.0 ± 9.8; 4.4–13.6		16.3 ± 13.1; 10.2–22.5		-		-		-		0.103

Continuous parameters are presented as mean ± standard deviation with 95% confidence interval; DSC = dice similarity coefficient; ASSD = average symmetric surface distance.

Mean dGEMRIC index of manual segmentation was 569 ± 113 (516–622)ms and 566 ± 115 (512–619ms for automatic segmentations, resulting in a mean relative difference of − 1.0 ± 0.9 (0.6–1.5, *p* = 0.033)%.

### External set 1 test

Mean DSC of the “external set 1 test” was lower for cartilage (0.89 ± 0.02 [0.88–0.90]; *p* < 0.001) and labrum (0.71 ± 0.04 [0.69–0.73]; *p* < 0.001) compared to the internal test set. Mean volume of the manually/automatically segmented cartilage was 7627 ± 1632 (6863–8391) / 8458 ± 1831 (7601–9316)mm^3^, resulting in a greater relative difference in volume of 10.6 ± 5.2 (8.1–13.0)% compared to the internal data set (*p* < 0.001). Mean volume of the manually/automatically segmented labrum was 2285 ± 581 (2013– 2557) / 2262 ± 612 (1976– 2548)mm^3^, resulting in a mean relative difference in volume of 9.0 ± 9.8 (4.4–13.6)% which did not differ from the internal test set (overall *p* = 0.103).

### External set 2 retraining

The DSC of the “external set 2 retraining” was higher for cartilage (0.92 ± 0.02 [0.91–0.93]; *p* < 0.001) and labrum (0.77 ± 0.05 [0.74– 0.79]; *p* = 0.020) compared to the “external set 1 test”. Mean volume of the manually/automatically segmented cartilage was 8208 ± 1699 (7412–9003) / 8111 ± 1829 (7255–8976)mm^3^, resulting in a lower mean relative difference in volume of 4.9 ± 3.7 (3.2–6.7)% compared to “external set 1 test” (*p* = 0.003). Mean volume of the manually/automatically segmented labrum was 2416 ± 711 (2083–2748) / 2288 ± 5.4 (2052–2523)mm^3^, resulting in a mean relative difference in volume of 16.3 ± 13.1% (10.2–22.5)%, which was comparable to the “external set 1 test” (overall *p* = 0.103).

### Inter- and intra-rater agreement

Inter-rater agreement of manual segmentation of cartilage/labrum yielded a DSC of 0.93 ± 0.04 (0.92–0.95) / 0.82 ± 0.05 (0.80–0.85) and a mean relative difference in volume of 4.9 ± 4.7 (2.7–7.1)% / 11.2 ± 10.1 (6.4–15.9)%, all comparable to the internal test set (all *p* > 0.232).

Intra-rater agreement of manual segmentation of cartilage / labrum yielded a DSC of 0.94 ± 0.03 (0.93–0.96) / 0.84 ± 0.07 (0.81–0.87) and a mean relative difference in volume of 4.4 ± 4.3 (2.4–6.4)% /10.9 ± 9.3 (6.6–15.2)%.

## Discussion

We demonstrated the feasibility of a fully automatic MRI segmentation method for hip cartilage and labrum with high performance for cartilage and labrum (mean DSC of 0.92 and 0.83, respectively; Tables 3 and 4) which was comparable to inter-rater agreement (mean DSC 0.93 for cartilage and 0.82 for labrum; *p* = 0.232 and 0.297, respectively). Model performance on the external data set 1 decreased for cartilage (mean DSC from 0.92 to 0.89; *p* < 0.001) and labrum (mean DSC from 0.83 to 0.71; *p* < 0.001; Tables 3 and 4). Providing the first external data set for additional training yielded a performance boost in the external data set 2 [external set 2 retrain for both cartilage (mean DSC of 0.92; *p* < 0.001) and labrum (mean DSC of 0.77; *p* = 0.020; Tables 3 and 4)]. This indicates the potential of increasing model performance using a small training set for application of the presented deep learning approach to a previously unknown pulse sequence.

**Fig. 5:**
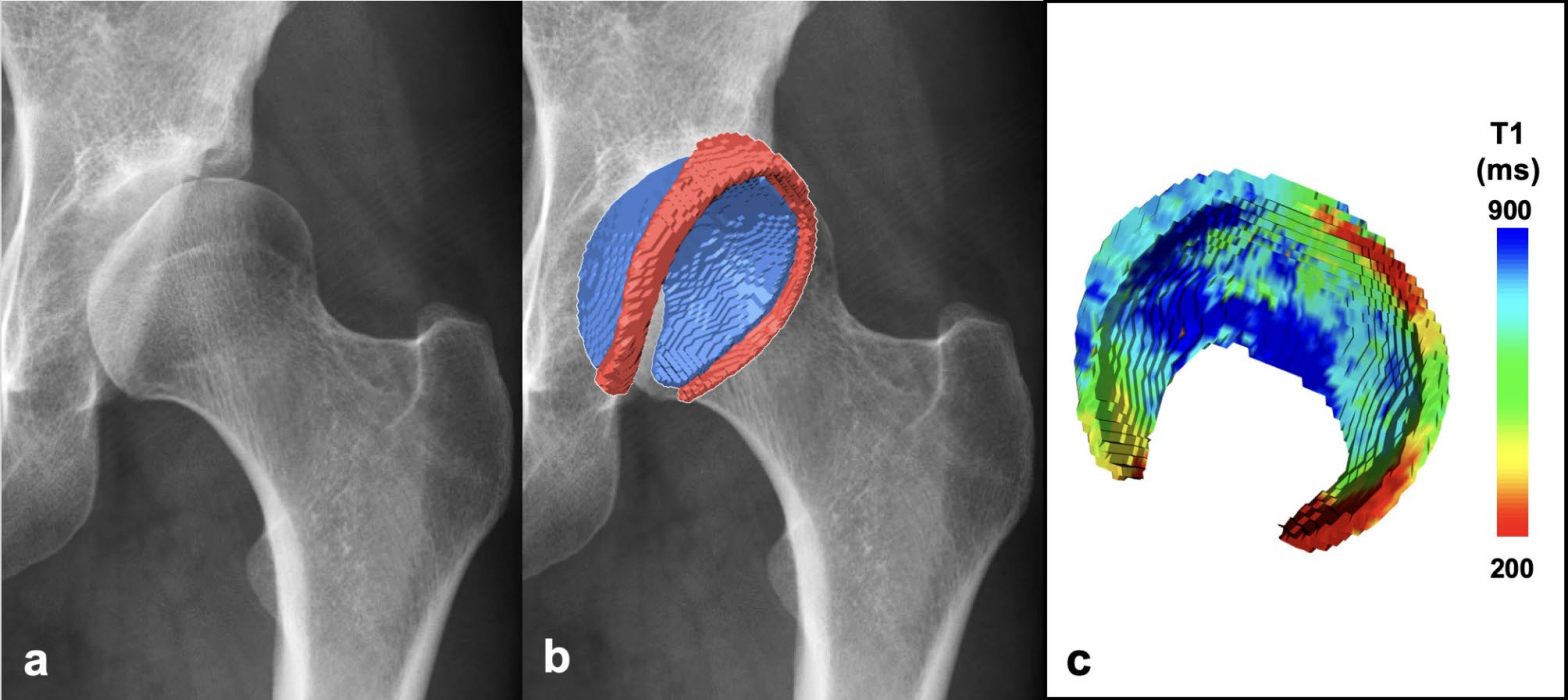
Patient example. (**a**) Antero-posterior radiograph of a 29 year old patient with hip dysplasia. (**b**) 3D morphologic models of hip cartilage and labrum demonstrate a large labrum volume with 1998 mm^3^ (cartilage volume = 5941 mm^3^, relative labrum volume = 34%), indicating labrum hypertrophy to compensate for deficient acetabular coverage. (**c**) 3D cartilage model with color coded T1 mapping demonstrate the typical dysplastic wear pattern of peripheral joint damage antero-superiorly indicated with lower T1 relaxation time (red color). 3D models were visualized using a custom-made software application for automatic segmentation and visualization^8^.

**Fig. 6:**
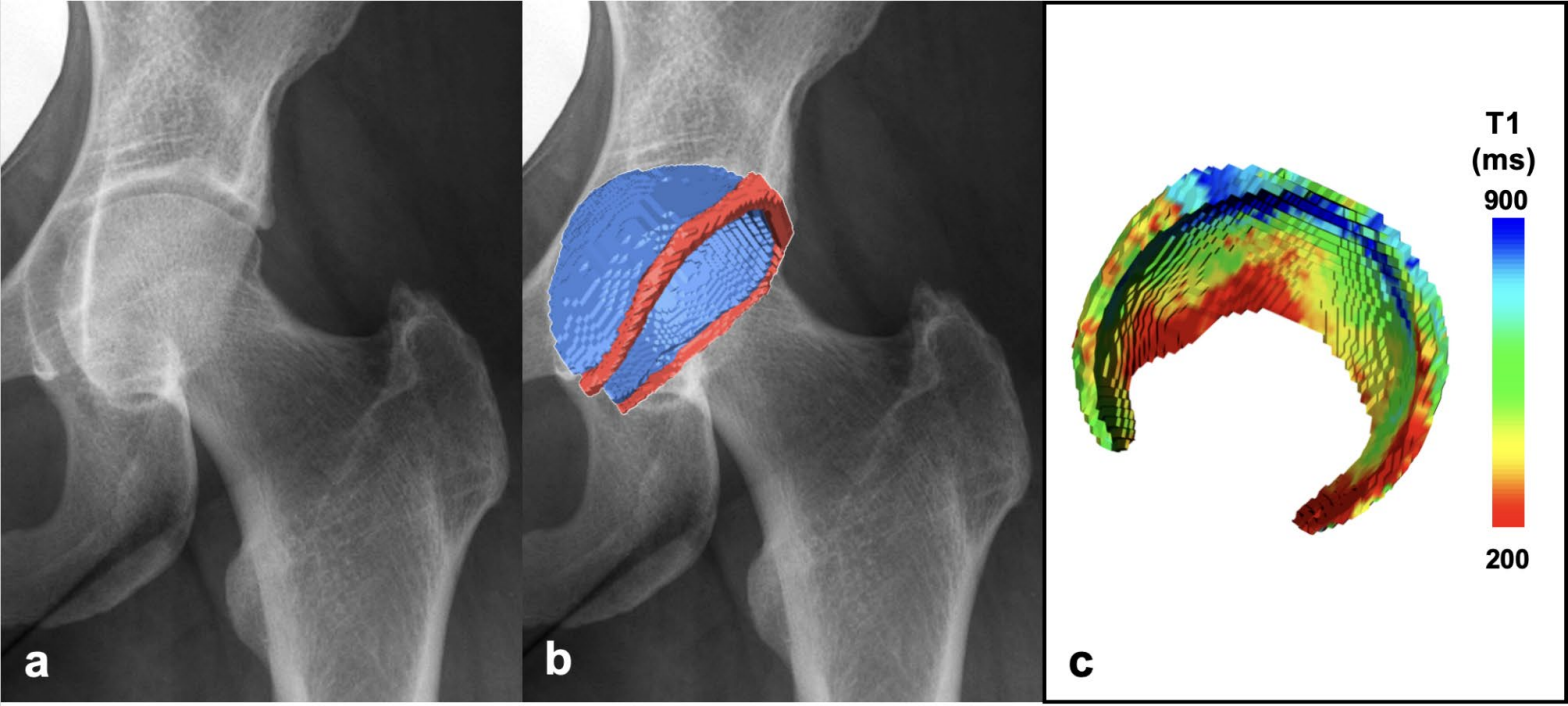
Patient example. (**a**) Antero-posterior radiograph of a 31 year old patient with pincer morphology. (**b**) 3D morphologic model of hip cartilage and labrum demonstrate a small labrum with a volume of 1043 mm^3^, (cartilage volume = 5438 mm^3^, relative labrum volume = 19%), indicating labrum hypotrophy as a result of global acetabular overcoverage. (**c**) 3D cartilage model with color coded T1 mapping demonstrates the typical pincer wear pattern of central joint degeneration. 3D models were visualized using a custom-made software application for automatic segmentation and visualization^8^. damage.

Our findings of automatic cartilage segmentation are in line with previous pilot studies in 25 hips for cartilage segmentation which yielded a slightly lower mean DSC of 0.92 using nnU-net^14^ or 0.86 using Latent3D-net^7^ based on a dual-flip angle based T1 cartilage mapping sequence. To bypass the problems related to B1 inhomogeneity when performing post-contrast mapping on 3T, we used a more robust technique (MP2RAGE)^15^ which yielded a clinically negligible relative bias of 1% (*p* = 0.033) in dGEMRIC indices between manual and automatic segmentations (Table 3). Instead of manual region of interest placement on selected 2D images, automatic volumetric assessment of cartilage quality paves way for large scale studies with the goal to define a prognostic threshold for T1 mapping of hip cartilage to improve patient selection for joint preserving surgery. Furthermore 3D visualization of cartilage quality using mapping techniques such as post contrast T1 mapping may enable further visualization of damage pattern which are not visible with conventional MRI such as medial wear pattern in global pincer FAI vs. a more peripheral damage pattern as seen in hip dysplasia^9^ (Figs. 5 and 6).

In contrast to our study, aforementioned reports^7,14^ did not assess model performance on unseen data or external datasets. In our study segmentation performance was high (mean DSC of 0.89 for cartilage and 0.71 for labrum) when applying the deep learning approach to an unseen morphologic 3D sequence (T2-w TrueFISP). This is important as high-resolution 3D sequences for evaluation of cartilage and labrum are commonly acquired in most institutions as part of the routine workup. Volumetric analysis of cartilage thickness yields great potential to improve the moderate prognostic value which has been demonstrated for indirect assessment of cartilage thickness based on evaluation of radiographic joint space narrowing^16,17^.

Labrum size and morphology reflect the underlying pathomechanism in the hip joint^9,10^ (Figs. 5 and 6). Therefore, there is great interest in finding ways to quantify differences in labrum size between different hip deformities which are difficult to assess using standard MRI evaluation. More specifically, the presence of labrum hypertrophy may support indication for pelvic osteotomy in patients with borderline hip dysplasia. Previous reports on 3D labrum segmentation included manual annotation of the labrum either on few selected reformatted radial images^18^ or on CT arthrograms^19^. In these studies, no attempts were made to automatize the process of labrum segmentation, which prevents large scale morphologic analysis of 3D morphology for clinical routine. To the best of the authors knowledge, this is the first study demonstrating the feasibility of automatic 3D segmentation of the hip labrum. In absence of previous reports in the literature, we compared the model performance to inter-rater agreement and found no difference.

This study has several limitations. Despite including a wide range of osseous hip deformities (Table 2), the sample size precludes subgroup analysis. We only included data from two sites (1.5T and 3T scanner) applying the model to two different pulse sequences from one vendor. Accordingly, larger multicentric and multivendor studies are needed to confirm generalizability of the presented deep learning approach. In addition, the presented results are based on direct MR arthrography only as we did not attempt to perform cartilage and labrum segmentation based on non-contrast MRI of the hip. In our institution direct MR arthrography of the hip is the preferred modality since capsular distension provided by intra-articular contrast better outlines the labrum from the adjacent peripheral capsular recess. Accordingly, we cannot extrapolate our findings to non-contrast MRI of the hip.

This study demonstrated high model performance for automatic segmentation of hip labrum and cartilage based on direct MR arthrography using 3D sequences. Automatic 3D analysis of chondro-labral morphology and cartilage composition paves way for large scale morphologic and prognostic studies which have great potential to improve surgical decision making by enhanced identification of the underlying hip pathomechanism and by more accurate prediction of joint damage.

## Data Availability

The code for preprocessing, training and evaluation is publicly available on GitHub. https://doi.org/10.5281/zenodo.14316889.
